# *In Silico* Study of Liquid Smoke Rice Husk against COVID-19

**DOI:** 10.1055/s-0042-1750776

**Published:** 2022-09-08

**Authors:** Ira Arundina, Neni Frimayanti, Meircurius D. C. Surboyo, Theresia I. Budhy, Benni Iskandar, Arya Pradana, Tytania Rahmaputry

**Affiliations:** 1 Department of Oral Biology, Faculty of Dental Medicine, Universitas Airlangga, Surabaya, Indonesia; 2 Sekolah Tinggi Ilmu Farmasi, Pekanbaru, Riau, Indonesia; 3 Department of Oral Medicine, Faculty of Dental Medicine, Universitas Airlangga, Surabaya, Indonesia; 4 Department of Oral Pathology and Maxillofacial, Faculty of Dental Medicine, Universitas Airlangga, Surabaya, Indonesia; 5 School of Pharmacy, College of Pharmacy, Taipei Medical University, Taipei, Taiwan; 6 Bachelor Dental Science Program, Faculty of Dental Medicine, Universitas Airlangga, Surabaya, Indonesia

**Keywords:** liquid smoke, rice husk, coronavirus disease 2019, infectious disease, *in silico*

## Abstract

**Objectives** Liquid smoke rice husk has been researched and proved to consist of antibacterial, analgesic, anti-inflammatory, and growth factor stimulatory properties. By these complex properties, the liquid smoke rice hull is able to purpose as a novel coronavirus disease 2019 (COVID-19) inhibitor. The research was conducted to analyze the role of the dominant compound in rice husk liquid smoke against one of the main proteases in complex with inhibitor N3 of COVID-19 and 6LU7 protein data bank (PDB) ID.

**Materialand Methods** The Molecular Operating Environment (MOE) 2020.0901 (Chemical computing group) was used to analyzed the interaction. The molecular structure test, including phenol, mequinol, 2-methoxy-phenol, 6-octadecenoic acid, oleic acid, 9-cctadecenoic acid, was chosen. The lopinavir as positive control and 6LU7 as COVID-19 protein were chosen. All the protein analyses were conducted using docking molecular.

**Result** The phenol, 2-methoxy-phenol, mequinol and 9-octadecenoic acid have higher binding free energy that causes difficult to bind to the active site of protein 6LU7 (−3.4758, −3.5509, −3.6845, and −5.0173 kcal/mol, respectively). The minor component of liquid smoke, such as 6-octadecenoic acid and oleic acid, has the binding free energy (−5,5327 and −5,4208 kcal/mol) and more factor of binding presumably as active COVID-19 inhibitor.

**Conclusion** The liquid smoke rice husk has active component like 6-octadecenoic acid and oleic acid are presumably as active COVID-19 inhibitor.

## Introduction

During the novel coronavirus disease 2019 (COVID-19) pandemic, various efforts have been made, both in prevention and treatment. 1 Various drugs have been used and developed as an effort to fight the virus that causes COVID-19. One of the ingredients takes part that is herbal remedies. Indonesia as one of the countries that has a high incidence rate, the use of herbal ingredients for the treatment and prevention of COVID-19 is widely used by the community. 2 3

Liquid smoke is a product of the process of breaking wood structures with high temperature. 4 Several ingredients in Indonesia that can be used to produce liquid smoke include rice husks, 5 coconut shells, 6 7 durian, 8 lamtoro, corn comb, 9 bamboo, 10 and cocoa bean skin. 11 Of these various types of materials, rice husks have been shown to have the potential to treat various diseases, including periodontitis, 12 oral ulcer, 13 14 15 16 diabetes, 17 18 and burn. 19 The liquid smoke rice hull contains phenol, 2-methoxy-phenol, mequinol, 6-octadecenoic acid, oleic acid, and 9-octadecenoic acid 5 that possess the analgesic, 20 anti-inflammation, 14 antibacterial, 12 21 and lower toxicity. 5 22 The liquid smoked rice husk is widely used by Indonesian as natural preservative for fish 23 24 and meat. 25 Due to the various potentials and uses of liquid smoke rice husk, it is necessary to carry out this potential for this liquid smoke, therefore an *in silico* analysis of the potential for COVID-19 is necessary. The research conducted was to analyze the role of the dominant compound in rice husk liquid smoke against one of the main proteases in complex with inhibitor N3 of COVID-19 and 6LU7.

## Material and Methods

### Ligand Preparation

**Fig. 1:**
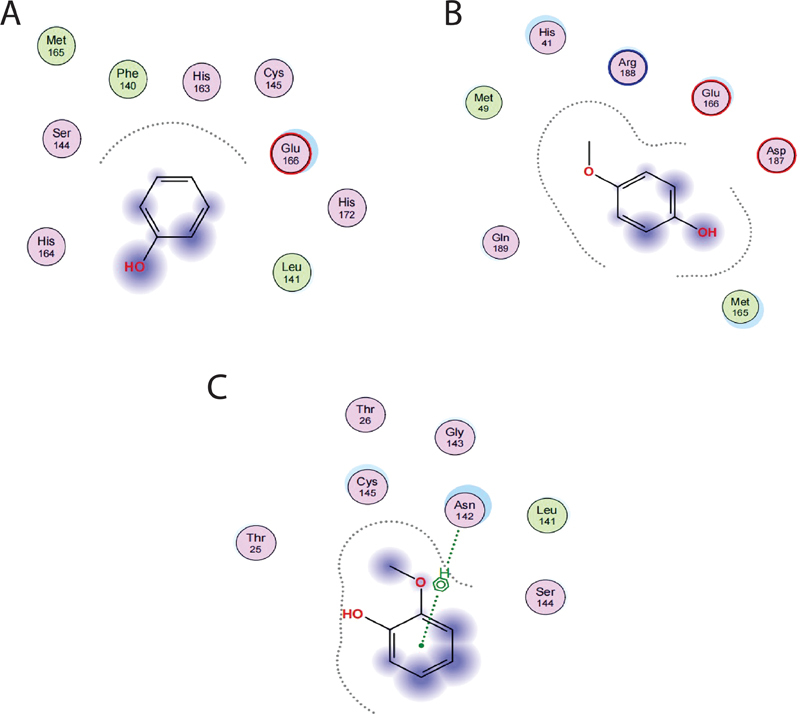
Spatial arrangement of phenol ( **A** ), mequinol ( **B** ), and 2-methoxy phenol ( **C** ).

The molecular structure of ligands, that is, phenol, mequinol, 2-methoxy-phenol, 6-octadecenoic acid, oleic acid, 9-octadecenoic acid, and lopinavir as positive control, were sketched using Chemdraw Professional 15.0 and saved in “.cdx” format, then the three-dimensional (3D) structure was further prepared using the Molecular Operating Environment (MOE) program 2020.0901 with MMFF94x force field and 0.0001 gradient. Then it is saved in *mdb format. Fig. 1 is presented the molecular structure of ligands.

### Protein Preparation

The protein structure used (PDB code: 6LU7) was downloaded from the web site ( www.rcsb.org ) in *PDB format. The crystal structure of this protein was then prepared using the application of discovery studio visualizer (DSV) 2020 and molecular operating environment (MOE) 2020.0901. Water molecules and native ligands were removed from the protein. Then the protein molecular structure was prepared using the MOE 2020.0901 software package. Next, CHARMM27 was selected as a force field, the protein was prepared with parameter, that is, RMS gradient was set to 0.01 kcal/mol/A. Energy minimization is performed on H atoms, α carbon, and also for backbone atoms. 26 The prepared structure is then saved in PDB format for then, and it can be used as a receptor for the docking process.

### Molecular Docking

The MOE 2020.0901 (Chemical computing group) was used to analyzed the interaction. Prior to docking, the active site of the protein was determined using a site finder, which consists of several amino acid residues, is then set as a dummy atom to serve as the target side for the docking process. Then in the dock menu, the site is set as a dummy atom and the MDB file containing the prepared ligand structure is selected as the ligand. Next, the placement is set as a triangle, the refinement is set as rigid and the pose is set as 50 and 10, respectively. After selecting the folder where the docking results are saved, click “Run” and wait until the docking process is complete.

## Result

Lopinavir as a positive control has the binding free energy of −9.8934 kcal/mol and an root mean square deviation (RMSD) value of 1.6436. In addition, lopinavir was able to form two hydrogen bonds with two amino acid residues, Met165 and Gln189. Lopinavir was also able to bind with Glu166 and Asp187 through the van der Waals interaction.

Based on the docking results, phenol was found the binding free energy value of −3.4758 kcal/mol and an RMSD value of 1.4211 ( Table 1 ). Thus, it indicated that the compound phenol is more difficult to bind to the active site of protein 6LU7. Based on the visualization of the docking results, it was observed that there are no hydrogen bond and hydrophobics interaction formed between the phenol and the active site 6LU7. Phenol only can form the van der Waals interaction with amino acid residue Glu166. The same case was with mequinol that did not construct any hydrogen bond; however, this compound was constructed the hydrophobic interaction through Arg188 and the van der Waals via Glu166 and Asp187. Unfortunately, mequinol has higher binding free energy value of −3.6845 kcal/mol with only six factors of binding. Thus, its makes phenol and mequinol become not potential as COVID-19 inhibitor.

**Table 1:** Docking results of liquid smoke rice husk with COVID-19

Compound	S (kcal/mol)	RMSD	H-bond	Hydrophobic	van der walls	The others interaction	Binding factor
Lopinavir (pose 2)	–9,8394	1.6436	Met165 Gln189	Arg188	Glu166 Asp187	Cys145, His164, Gly143, His41, Thr190, Ser46, Thr24, Thr26, Thr25, Asn142, Ser144, Leu141, Met49, Ala191, Leu27	20
Phenol	–3,4758	1.4211	–	–	Glu166	Met165, Phe140, Ser144, His164, Leu141, His172, Cys145, His163,	6
Mequinol	–3,6845	0.6694	–	Arg188	Glu166 Asp187	Met49, Gln189, Met165, His41	7
2-methoxy-phenol	–3,5509	1.6702	Asn142	–	–	Thr25, Ser144, Leu141, Gly143, Cys145, Thr26	7
6-octadecenoic acid	–5,5327	1.2351	–	Ser144 Arg188	–	Cys145, Gly143, Leu141, Asn142, His41, Met165, Gln189, Ser46, Thr24, Thr26, Met49, Thr25, Leu27	15
9-octadecenoic acid	–5,0713	1.2453	–	–	Glu166	Met165, His172, His164, His163, Phe140, Asn142, Thr25, Leu141, Thr26, Gly143, Ser144, Cys145	10
Oleic acid	–5,4208	1.2288	Thr24	–	Glu166	Ser144, Gly143, Cys145, Asn142, His163, Leu141, His172, His164, Phe140, Met165, Cys44, Thr45, Thr25, Ser46, Thr26, Met49	13

Abbreviations: COVID-19, novel coronavirus 2019; RMSD, root mean square deviation.

The 2-methoxy-phenol has the binding-free energy value of −3.5509 kcal/mol. This compound was performed a hydrogen bond between methoxy group of ligands with amino acid residue Asn142, but there is no interaction with hydrophobic and the van der Waals. The 9-octadecenoic acid has binding-free energy of −5.0173 kcal/mol. It has the van der Waals interaction with amino acid residue Glu166. Furthermore, these compounds become not potential as COVID-19 inhibitor. The spatial arrangement of these compounds is depicted in Fig. 1 .

**Fig. 2:**
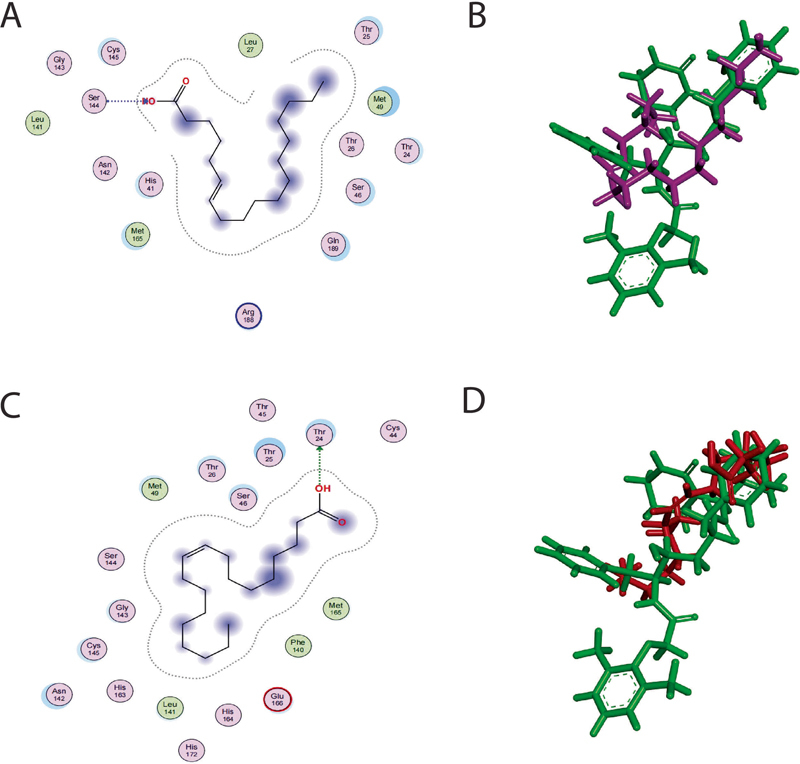
Spatial arrangement and superimpose of 6-octadenoic acid (purple) ( **A** and **B** ) and oleic acid (red) ( **C** and **D** ) with lopinavir (green).

The 6-octadecenoic acid and oleic acid are presumably as active COVID-19 inhibitor, because these components have the binding free energy of −5,5327 and −5,4208 kcal/mol, respectively. in addition, these compounds have more factor of binding than the other compounds. Factor of binding is the probability for the occurrence of receptor–ligand binding to the same amino acid residues with the positive control. Thus, it makes these compounds become has a potential against COVID-19. The spatial arrangement and superimpose of these of compounds with lopinavir are depicted in Fig. 2 .

According to docking results, superimposition of 6-octadenoic acid (purple) and oleic acid (red) with lopinavir (green) seem that 6-octadenoic acid and oleic acid have the same orientation with lopinavir to bind with protein 6LU7. Although 6-octadenoic acid has binding-free energy higher than lopinavir. Thus, it makes 6-octadenoic acid become potential against COVID-19.

## Discussion

The virus that causes COVID-19, namely, severe acute respiratory syndrome-coronavirus-2 (SARS-CoV-2) encodes two proteins, namely, pp1a and pp1ab, which are functional polypeptides that play an important role in the process of replication and transcription. This polypeptide is released by the catalytic cleavage activity of 3-chymotrypsin-like cystein (3CL). The structure of 3CL is known and is present in a complex with an N3 inhibitor known as 6LU7 in PBD. 27 This structure is the target of drugs used to inhibit the COVID-19 virus, one of which is lapinavir. 28 This drug was originally used for HIV therapy as a protease inhibitor. 29 The protease inhibitor is very important used to prevent the virus from replicating.

Rice husk liquid smoke has been known to have 28 components. The 2-methoxy-phenol, mequinol, phenol, 6-octadecenoic acid, oleic acid, and 9-octadecenoic acid are the dominant components in which there is liquid smoke of rice husk. 5 Only two from six compounds in the liquid smoke rice husk determined as active inhibitor for 6LU7. The two component was 6-octadecenoic acid and oleic acid. Bot component has proportion as 7.81%, using gas chromatograph mass spectrometry. 5 These component determined as active inhibitor because the component have a higher binding factors. The 6-octadecenoic acid has 15 binding factor including Cys145, Gly143, Leu141, Asn142, His41, Met165, Gln189, Ser46, Thr24, Thr26, Met49, Thr25, Leu27, Ser144, and Arg188. And the oleic acid has 13 binding factors to Ser144, Gly143, Cys145, Asn142, Leu141, His164, Met165, Thr25, Ser46, Thr26, Met49, Thr24, and Glu166.

Other factor determined the component as active inhibitor was the free-binding capacity and the RMSD value. The 6-octadecenoic acid and oleic acid has higher RSMD (1.2453 and 1.2288) and the binding free capacity was −5,5327 and −5,4208 kcal/mol.

Both components are predicted to have the ability to inhibit COVID-19 but there are no clinical trials that prove this. The potential of rice husk liquid smoke to treat various pathological conditions has been investigated including periodontitis, 12 oral ulcer, 13 14 15 16 diabetes, 17 18 and burn. 19 However, these studies have only been performed on experimental animals and have not been performed in clinical trials or in humans. This is a challenge to be able to imply the potential of rice husk liquid smoke as a candidate for a COVID-19 inhibitor.

## Conclusion

Only two components presumably work as active COVID-19 inhibitor which are 6-octadecenoic acid and oleic acid.
